# Mmp12 Is Translationally Regulated in Macrophages during the Course of Inflammation

**DOI:** 10.3390/ijms242316981

**Published:** 2023-11-30

**Authors:** Silvia Kuntschar, Giulia Cardamone, Kevin Klann, Rebekka Bauer, Sofie Patrizia Meyer, Rebecca Raue, Peter Rappl, Christian Münch, Bernhard Brüne, Tobias Schmid

**Affiliations:** 1 Institute of Biochemistry I, Faculty of Medicine, Goethe University Frankfurt, 60590 Frankfurt, Germany; 2 Institute of Biochemistry II, Faculty of Medicine, Goethe University Frankfurt, 60590 Frankfurt, Germany; 3 Frankfurt Cancer Institute, Goethe University Frankfurt, 60596 Frankfurt, Germany; 4 German Cancer Consortium (DKTK), Partner Site Frankfurt, 60590 Frankfurt, Germany; 5 Fraunhofer Institute for Translational Medicine and Pharmacology, 60596 Frankfurt, Germany

**Keywords:** macrophage, inflammation, efferocytosis, Mmp12, translation, resolution

## Abstract

Despite the importance of rapid adaptive responses in the course of inflammation and the notion that post-transcriptional regulation plays an important role herein, relevant translational alterations, especially during the resolution phase, remain largely elusive. In the present study, we analyzed translational changes in inflammatory bone marrow-derived macrophages upon resolution-promoting efferocytosis. Total RNA-sequencing confirmed that apoptotic cell phagocytosis induced a pro-resolution signature in LPS/IFNγ-stimulated macrophages (Mϕ). While inflammation-dependent transcriptional changes were relatively small between efferocytic and non-efferocytic Mϕ; considerable differences were observed at the level of de novo synthesized proteins. Interestingly, translationally regulated targets in response to inflammatory stimuli were mostly downregulated, with only minimal impact of efferocytosis. Amongst these targets, pro-resolving matrix metallopeptidase 12 (Mmp12) was identified as a translationally repressed candidate during early inflammation that recovered during the resolution phase. Functionally, reduced MMP12 production enhanced matrix-dependent migration of Mϕ. Conclusively, translational control of MMP12 emerged as an efficient strategy to alter the migratory properties of Mϕ throughout the inflammatory response, enabling Mϕ migration within the early inflammatory phase while restricting migration during the resolution phase.

## 1. Introduction

While inflammation is an important response to infection or injury [1], its efficient resolution is critical to prevent excessive damage due to chronic inflammatory conditions and to restore functional homeostasis [2,3]. Acute inflammatory reactions are characterized by the production of inflammatory mediators by resident cells and subsequent infiltration of polymorphonuclear cells (PMNs), primarily neutrophils, to the site of inflammation [4]. Within the highly reactive inflammatory microenvironment, PMNs undergo apoptosis and are cleared via efferocytosis by tissue-resident and recruited monocyte-derived macrophages (Mϕ) [5,6,7]. Their function is to phagocytose and eliminate pathogens and tissue debris. Efferocytosis further contributes to the reprogramming of macrophages from a pro-inflammatory to a pro-resolution state and, thus, represents an important trigger for initiating the inflammatory resolution phase [8,9,10,11,12,13].

While many of the acute, pro-inflammatory responses are regulated at the transcription level, post-transcriptional modes of regulation contribute to fine-tuning, gaining importance, especially during later stages of the inflammatory response [14,15,16]. Noteworthy, translational regulation has emerged as a key node modulating gene expression in immune cells [17]. Moreover, translation-associated gene expression signatures are massively enriched in interleukin 4 (IL-4)-treated, anti-inflammatory Mϕ [18], while the down-regulated genes are enriched in translation when resolution is attenuated in Mϕ [19]. Furthermore, changes in gene expression in tumor-associated macrophages (TAMs) during the shift from a pro- to an anti-inflammatory phenotype are predominantly modulated via selective changes in translation rather than transcription [20].

In the present study, we identified the resolution-phase associated matrix metallopeptidase 12 (Mmp12) as a novel translationally regulated target in Mϕ during inflammation and characterized the associated Mϕ migration-regulatory properties.

## 2. Results

While efferocytosis is critical to achieving efficient inflammation resolution, and adaptive changes during inflammation, especially during later phases, are commonly regulated at a post-transcriptional level, translational changes in Mϕ induced by efferocytosis remain largely elusive. We recently reported that translation is differentially affected when resolution is altered in a murine peritonitis model [19]. Therefore, we set out to determine the impact of efferocytosis on translational changes throughout the course of inflammation.

### 2.1. Efferocytosis Alters Inflammatory Responses in Macrophages

**Figure 1:**
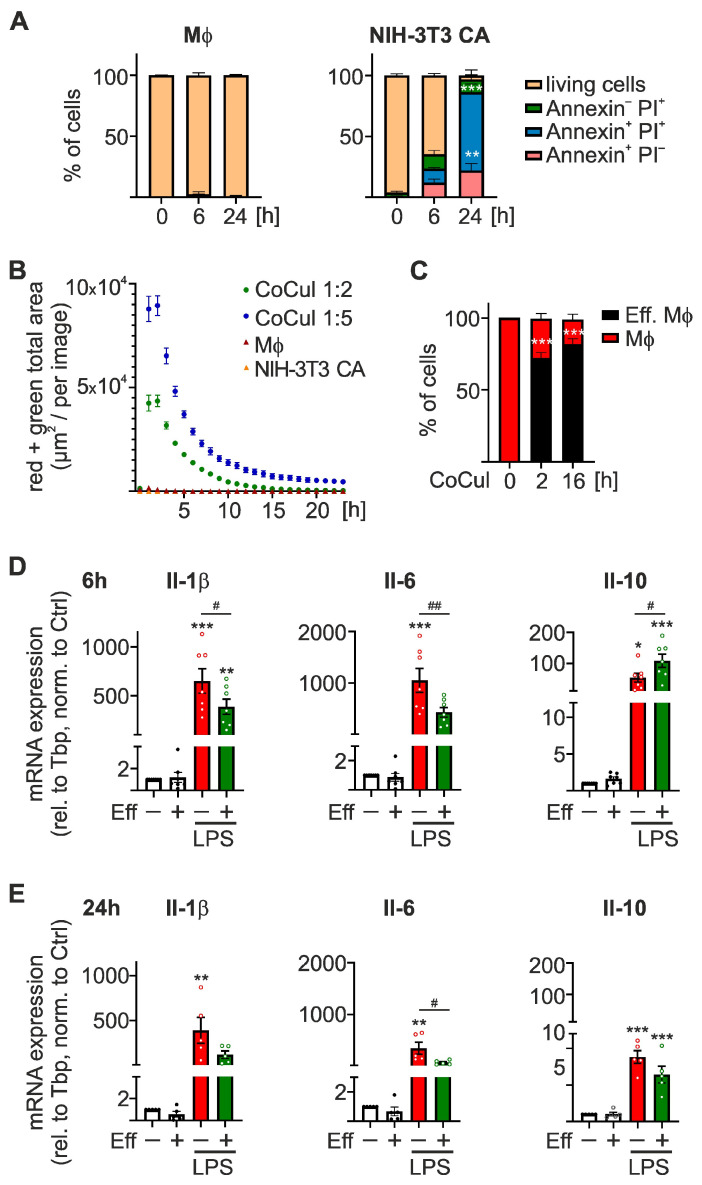
Impact of efferocytosis on inflammatory responses in Mϕ. (**A**) Bone marrow-derived Mϕ (BMDMs) and NIH-3T3 caspase activatable (CA) cells were treated with 10 nM dimerizer for 6 and 24 h, stained with Annexin V and propidium iodide (PI), and analyzed using flow cytometry (*n* = 3). (**B**) BMDMs were stained with MitoTracker red, and apoptotic NIH-3T3 CA cells (after 6 h treatment with 10 nM dimerizer) were stained with pHrodo for 1 h prior to co-culture at a 1:2 or 1:5 ratio. Efferocytosis was followed by tracking double-positive cells in an Incucyte live cell analysis system for 24 h (*n* = 3). (**C**) BMDMs were stained with MitoTracker red, and apoptotic NIH-3T3 CA cells (after a 6 h treatment with 10 nM dimerizer) were stained with CFSE prior to co-culture at a 1:5 ratio for 2 or 16 h. Efferocytic Mϕ were assessed by flow cytometry (*n* = 3). (**D**,**E**) BMDMs were co-cultured with or without apoptotic NIH-3T3 CA cells (Eff) (after 6 h dimerizer treatment) at a 1:5 ratio for 16 h, prior to stimulation with 100 ng/mL LPS and 100 U/mL IFNγ for 6 h (**D**) or 24 h (**E**) (LPS). CD45^+^ Mϕ were purified by magnetic-activated cell sorting (MACS) and mRNA expression was quantified by RT-qPCR analysis. mRNA expression was normalized to Tbp and is given relative to untreated control Mϕ (*n* ≥ 5). Data are presented as means ± SEM and were statistically analyzed using two-way ANOVA with Tukey’s multiple comparisons test; * *p* < 0.05, ** *p* < 0.01, *** *p* < 0.001 compared to untreated control Mϕ; ^#^ *p* < 0.05, ^##^ *p* < 0.01 compared to LPS/IFNγ-treated control Mϕ.

To gain insights into translational changes in Mϕ induced by efferocytosis, we initially established an in vitro murine efferocytosis model. To this end, a genetically modified murine NIH-3T3 caspase-8 activatable (CA) fibroblast cell line was used, which stably expresses a dimerizable human caspase-8 domain and, thus, was shown to undergo selective caspase-8-dependent apoptosis upon treatment with a B/B Homodimerizer (dimerizer) [21]. As expected, dimerizer (10 nM) treatment efficiently induced apoptotic and secondary necrotic cell death in CA cells. Within 6 h of adding the dimerizer, 12.1 ± 2.7% of the cells were apoptotic (Annexin V^+^ PI^−^), and 11.5 ± 0.9% were necrotic (Annexin V^+^ PI^+^) (Figure 1A, *right panel*), while after 24 h most fibroblasts appeared necrotic (64.2 ± 6.8%). In contrast, bone-marrow-derived Mϕ (BMDMs) remained unaffected by dimerizer treatment (Figure 1A, *left panel*). Since phagocytosis of apoptotic cells induces alternative Mϕ phenotypes [8], 6 h dimerizer pre-treatment was used to induce apoptosis in NIH-3T3 CA cells for subsequent co-culture with BMDMs to characterize efferocytosis-dependent changes in Mϕ translation. To determine the efferocytosis capacity of BMDMs, we labeled NIH-3T3 CA cells with pHrodo. This dye emits a green fluorescent signal only within a low pH environment, such as the lysosomal compartment of phagocytes. When co-culturing MitoTracker red-labeled Mϕ with pHrodo-labeled apoptotic cells, double-positive Mϕ were considered to actively efferocytose and lysosomally degrade apoptotic cells. Live cell imaging analyses revealed that Mϕ rapidly take up apoptotic cells, reaching maximal levels after 2 h (Figure 1B). Increasing the proportion of apoptotic cells within the co-culture from 2- to 5-fold relative to Mϕ further led to a higher uptake of apoptotic cells, which suggests that Mϕ can rapidly take up substantial amounts of apoptotic cells. Additionally, while pHrodo-labeled apoptotic CA cells alone did not emit a green signal, co-culture samples displayed a strong green signal, demonstrating the necessity of the uptake by Mϕ (Figure S1).

While efferocytosis appeared to be extremely fast, flow cytometric analyses revealed that 81.8 ± 3.7% of all Mϕ contained apoptotic cell material after overnight co-culture (Figure 1C).

Having established the rapid efferocytosis process, which should limit the occurrence of secondary necrosis of the apoptotic cells, we next asked how efferocytosis affects Mϕ activation in our model. Therefore, we co-cultured BMDMs with 6 h dimerizer-treated NIH-3T3 CA cells at a 1:5 ratio for 16 h before stimulating washed Mϕ for an additional 6 or 24 h with LPS (100 ng/mL)/IFNγ (100 U/mL). In line with previous reports, efferocytosis markedly reduced LPS/IFNγ-induced expression of pro-inflammatory interleukin-1β (IL-1β) and IL-6 at 6 and 24 h. In contrast, efferocytosis enhanced IL-10 expression at 6 h of LPS treatment, leaving it unaltered at 24 h (Figure 1D,E). However, efferocytosis alone did not alter basal cytokine expression.

These findings suggest that efferocytosis of apoptotic cells by BMDMs might alter the kinetics of the inflammatory response to LPS/IFNγ, indicating an altered resolution phenotype.

### 2.2. Influence of Efferocytosis on Inflammatory mRNA Expression and Translation

**Figure 2:**
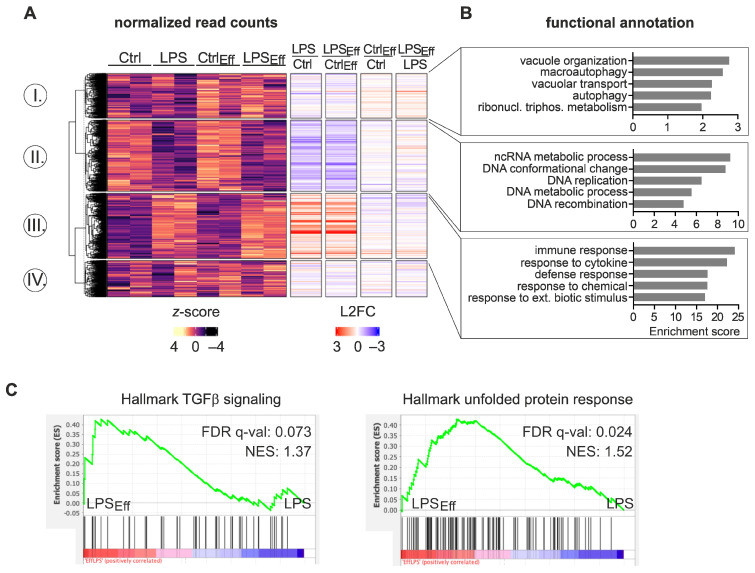
Differential mRNA expression changes in response to inflammatory stimulation between efferocytic and non-efferocytic Mϕ. BMDMs were co-cultured with or without apoptotic NIH-3T3 CA cells (Eff) (after 6 h dimerizer treatment) at a 1:5 ratio for 16 h prior to stimulation with 100 ng/mL LPS and 100 U/mL IFNγ for 6 h (LPS). CD45^+^ Mϕ were purified by MACS-sorting followed by total RNA-seq analysis (*n* = 2). (**A**) Normalized read counts of differentially expressed genes (DEGs) (padj < 0.05) were visualized in a heatmap (*z*-score normalized counts) and categorized into clusters I, II, III, and IV by *k*-means clustering. Annotation columns depict the log2 fold change (L2FC) of DEGs. (**B**) Top five functional annotation clusters for each cluster as identified by DAVID [23,24]. (**C**) Gene set enrichment analysis (GSEA) of LPS/IFNγ-stimulated naïve vs. efferocytic Mϕ (*p* < 0.1, FDR < 0.1; NES = normalized enrichment score).

To gain further insights into the impact of efferocytosis on inflammatory Mϕ responses, we performed total mRNA sequencing (RNA-seq) of untreated or 6 h LPS/IFNγ-treated Mϕ in the presence or absence of apoptotic cells. Principal component analysis (PCA) indicated that the main contributor to mRNA expression changes was LPS treatment (PC1: 77% variance), while efferocytosis appeared to account for PC2 (15% variance) (Figure S2). In total, 9589 differentially expressed genes (DEGs) (padj < 0.05) were found (Figure 2A; Table S1). While *k*-means clustering yielded a group of 1954 DEGs upregulated by efferocytosis independent of inflammatory stimulation (cluster I), no DEG group was identified as down-regulated by efferocytosis. In line with the PCA analysis, most changes appeared to be induced by LPS/IFNγ treatment. Specifically, 3163 DEGs were downregulated (cluster II), and 2867 DEGs were upregulated by inflammatory stimulation (cluster III), with fold changes similar in naïve and efferocytic Mϕ (*annotation columns*). Functionally, efferocytosis-induced DEG changes (cluster I) were strongly enriched for gene ontology (GO) terms associated with the efferocytosis process, such as autophagy and vacuole organization (Figure 2B; Table S2). In line with the close relationship between inflammation and metabolism, LPS/IFNγ-downregulated DEGs were enriched in metabolic processes, including ncRNA and DNA metabolic processes (cluster II). Meanwhile, LPS/IFNγ-induced DEGs showed a strong enrichment of immune system-associated terms (cluster III). Interestingly, although efferocytosis-induced changes contributed to a minor proportion of gene expression changes, gene set enrichment analysis (GSEA) revealed that in the inflammatory context, efferocytosis was also enriched in “TGFβ signaling”, suggestive of a resolution phenotype (Figure 2C, *left panel*; Table S3). Of note, inflammatory Mϕ efferocytosis appeared to further enrich the hallmark “unfolded protein response”, which alters translational processes [22].Thus, while efferocytosis induced a pro-resolution phenotype in LPS/IFNγ-stimulated Mϕ, most genes appeared to be similarly regulated between naïve and efferocytic Mϕ within the inflammatory context. 

**Figure 3:**
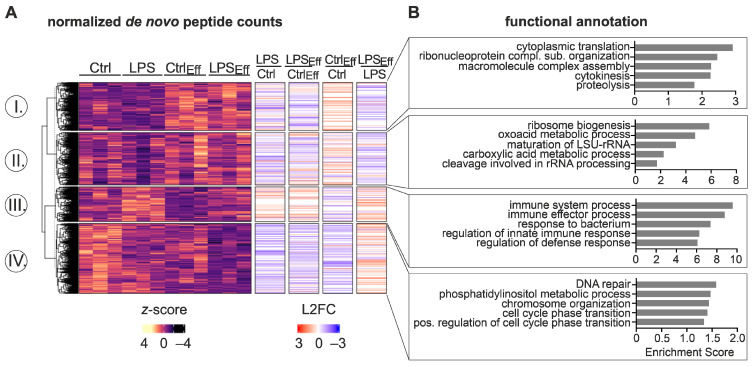
Differential de novo proteomic changes in response to inflammatory stimulation between efferocytic and non-efferocytic Mϕ. BMDMs were co-cultured with or without apoptotic NIH-3T3 CA cells (Eff) (after 6 h dimerizer treatment) at a 1:5 ratio for 16 h before stimulation with 100 ng/mL LPS and 100 U/mL IFNγ for 6 h (LPS). CD45^+^ Mϕ were purified by MACS-sorting followed by multiplexed enhanced protein dynamics proteomics (mePROD) (*n* = 3). (**A**) Normalized de novo peptide counts of differentially expressed peptides (DEPs) (padj < 0.05) were visualized in a heatmap (*z*-score normalized counts) and categorized into clusters I, II, III, and IV by *k*-means clustering. Annotation columns depict log2 fold change (L2FC) of DEPs. (**B**) Top five functional annotation clusters for each cluster as identified by DAVID [23,24].

To determine if inflammatory responses and efferocytosis alter Mϕ functioning at a post-transcriptional level, we used multiplexed enhanced protein dynamics proteomics (mePROD) [25]. In this way, we determined changes in de novo protein expression (i.e., translation) in Mϕ induced by efferocytosis and LPS/IFNγ stimulation. *k*-means clustering of the 4037 differentially expressed peptides (DEPs) (padj < 0.05) identified four groups distinguishable by differential regulatory patterns (Figure 3; Table S4). First, 949 candidates were translationally enhanced by efferocytosis with minimal impact of LPS/IFNγ (cluster I) (Figure 3A). This cluster was strongly enriched for translation-associated processes (Figure 3B; Table S5). The second group of targets (1035) was enriched in ribosome- and metabolism-associated terms and displayed reduced translation in response to LPS/IFNγ but showed generally higher DEP counts in efferocytic Mϕ (cluster II). A small proportion of the DEPs (685) appeared to be induced by LPS/IFNγ treatment in naïve Mϕ with lower translational levels in efferocytic Mϕ (cluster III). This cluster was further enriched for inflammation-associated processes. While candidates in the fourth cluster appeared to be translationally repressed in efferocytic Mϕ, their translation was reduced upon inflammatory stimulation (cluster IV). Functionally, this cluster was enriched for DNA repair and cell cycle-associated functions.

Taken together, changes between LPS/IFNγ-treated naïve and efferocytic Mϕ were much higher at the de novo proteomic (DEPs) level than at the mRNA expression (DEGs) level, suggesting a substantial contribution of translational regulation throughout the course of inflammation.

### 2.3. Characterization of Matrix Metallopeptidase 12 (Mmp12) Regulation throughout the Course of Inflammation

**Figure 4:**
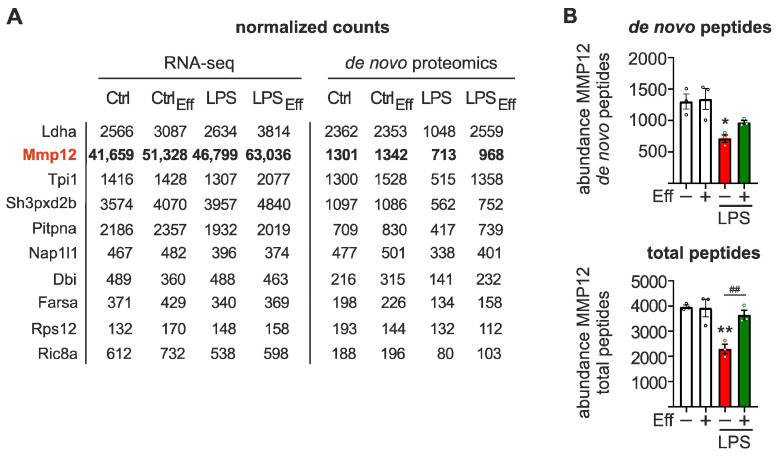
Selection of translationally regulated targets in inflammatory Mϕ. (**A**) For the selection of candidates predominantly regulated at the translation level upon inflammatory stimulation (100 ng/mL LPS and 100 U/mL IFNγ, 6 h), substantially expressed targets (normalized read/peptide counts > 50) were filtered for pronounced LPS/IFNγ-selective regulation of de novo proteins (|FC_Ctrl vs. LPS_| > 2; |FC_Ctrl vs. Eff_| < 1.5) with minimal regulation at the mRNA level (|FC_Ctrl vs. LPS_| < 1.2). Normalized read (*left columns*) and de novo peptide counts (*right columns*) of the top ten selected targets, sorted by Ctrl de novo peptide counts, are shown. (**B**) Abundance of MMP12 de novo peptides (*upper panel*) and total peptides (*lower panel*) based on de novo synthesis proteomics data. Data are presented as means ± SEM and were statistically analyzed using two-way ANOVA with Tukey’s multiple comparisons test; * *p* < 0.05, ** *p* < 0.01 compared to untreated control Mϕ; ^##^ *p* < 0.01 compared to untreated efferocytic Mϕ.

Having established global transcriptomic and de novo proteomic changes in inflammation and efferocytosis-associated resolution, we aimed to identify targets regulated specifically at the translation level during inflammation. Therefore, we selected candidates displaying strong regulation at the de novo proteomic level by LPS/IFNγ, with only minimal regulation at the mRNA expression level. Candidates with only minimal expression, as well as those responding to efferocytosis alone, were excluded. Interestingly, based on these selection criteria, we did not identify any candidate with increased translation; rather, all resulting targets showed reduced translation in Mϕ upon LPS/IFNγ stimulation (Figure 4A; Table S6). Of the resulting candidates, matrix metallopeptidase 12 (MMP12), also known as macrophage elastase, was the most highly expressed at the mRNA level without correspondingly high translation levels. The relatively low protein output, as compared to the extremely high mRNA levels, suggests a potential translation regulatory component. Notably, total MMP12 protein expression (Figure 4B, *lower panel*) corresponded with the observed translational output changes (Figure 4B, *upper panel*), suggesting that translational changes might contribute to functional protein levels.

**Figure 5:**
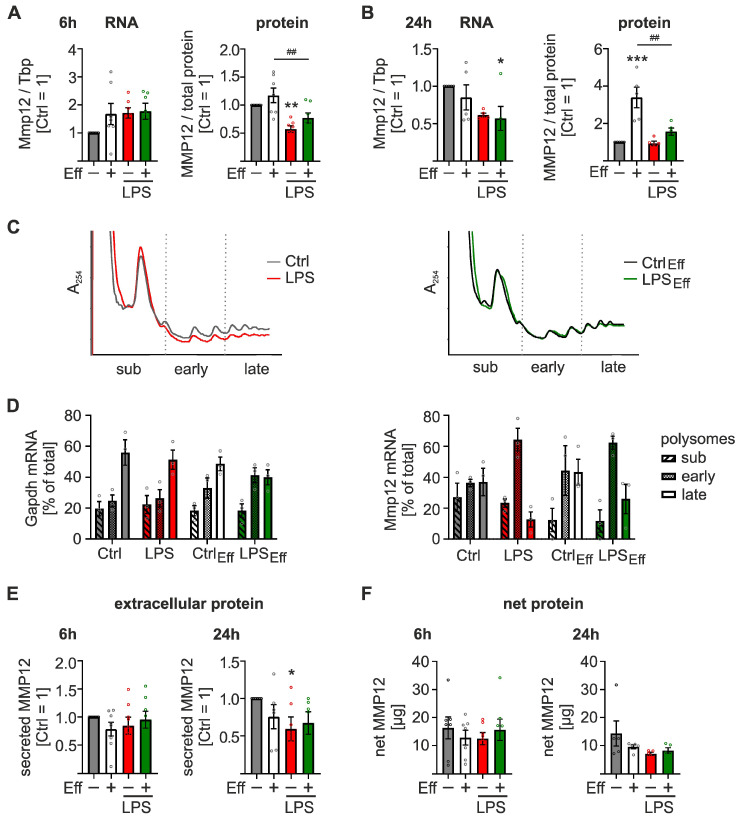
Translational regulation of matrix metallopeptidase 12 (Mmp12) in inflammatory Mϕ. BMDMs were co-cultured with or without apoptotic NIH-3T3 CA cells (Eff) (after 6 h dimerizer treatment) at a 1:5 ratio for 16 h prior to stimulation with 100 ng/mL LPS and 100 U/mL IFNγ for 6 or 24 h (LPS). For further analyses, CD45^+^ Mϕ were purified by MACS-sorting. (**A**) Mmp12 mRNA expression was quantified by RT-qPCR analysis, normalized to Tbp, and presented relative to untreated control Mϕ (*n* ≥ 5). (**B**) MMP12 protein expression was analyzed by western blot analysis, normalized to total protein, and presented relative to untreated control Mϕ (*n* ≥ 5). (**C**,**D**) Translational status of Mmp12 was assessed by polysomal fractionation analysis. (**C**) UV profiles identified sub-polysomal (sub), early, and late polysomal fractions (representative tracks of three independent experiments are shown). (**D**) Gapdh *(left panel*) and Mmp12 mRNA (*right panel*) distribution across the gradients was analyzed by RT-qPCR (*n* = 3). (**E**) Secreted MMP12 protein was quantified in Mϕ supernatants by ELISA and is presented relative to untreated control Mϕ (*n* ≥ 5). (**F**) Net protein expression of MMP12 was calculated by combining mean intra- and extracellular MMP12 protein expression and is presented relative to untreated control Mϕ (*n* ≥ 5). Data are presented as means ± SEM and were statistically analyzed using two-way ANOVA with Tukey’s multiple comparisons test; * *p* < 0.05, ** *p* < 0.01, *** *p* < 0.001 compared to untreated control Mϕ; ^##^ *p* < 0.01 compared to LPS/IFNγ-treated control Mϕ.

As MMP12 is important during inflammation resolution [26], we aimed to further characterize the mechanistic details of MMP12 regulation during inflammation. In line with the observations in the global data sets, Mmp12 mRNA expression remained largely unaltered (Figure 5A, *left panel*), and MMP12 protein was significantly downregulated after 6 h LPS/IFNγ stimulation, independent of the efferocytosis state (Figure 5A, *right panel*; Figure S3). In contrast, Mmp12 mRNA expression decreased 24 h after inflammatory stimulation (Figure 5B, *left panel*), while LPS/IFNγ treatment only attenuated the efferocytosis-dependent increase in MMP12 protein (Figure 5B, *right panel*; Figure S3). To investigate whether the discrepancy in mRNA and protein expression in inflammatory Mϕ was indeed due to translational changes, we used polysomal fractionation analyses to determine the translation efficiency of Mmp12 mRNA. Global translation appeared to be reduced after 6 h of LPS/IFNγ stimulation in naïve Mϕ, whereas no such effect was observed in efferocytic Mϕ (Figure 5C). While Gapdh mRNA predominantly resided in the late polysomal fractions, indicative of efficient translation, and remained unaffected by LPS/IFNγ treatment (Figure 5D, *left panel*), Mmp12 redistributed from late to early polysomal fractions, suggesting reduced translational efficiency upon inflammatory stimulation (Figure 5D, *right panel*). Interestingly, translation was also reduced in efferocytic Mϕ by LPS/IFNγ to a lesser extent, which corroborated the protein level observations (Figure 5A).

Since MMP12 exerts extracellular functions, we assessed extracellular MMP12 protein levels in the supernatants of naïve or efferocytic Mϕ upon inflammatory stimulation. In line with the intracellular MMP12 protein levels, extracellular MMP12 levels in Mϕ supernatants decreased upon LPS/IFNγ stimulation, though with a slight delay, i.e., after 24 h (Figure 5E).

The absolute quantification of net MMP12 protein levels (i.e., the combined intra- and extracellular levels) further supported a slight decrease in MMP12 after 6 h of LPS/IFNγ treatment, which became more pronounced after 24 h (Figure 5F). As predicted based on its extracellular function, most MMP12 (99.99972% +/− 0.00024%) appeared to be secreted in all samples, independent of the treatments.

In summary, these data suggest that the reduction in pro-resolving MMP12 protein during early inflammation is largely controlled translationally.

### 2.4. MMP12 Suppresses Migration of Macrophages

**Figure 6:**
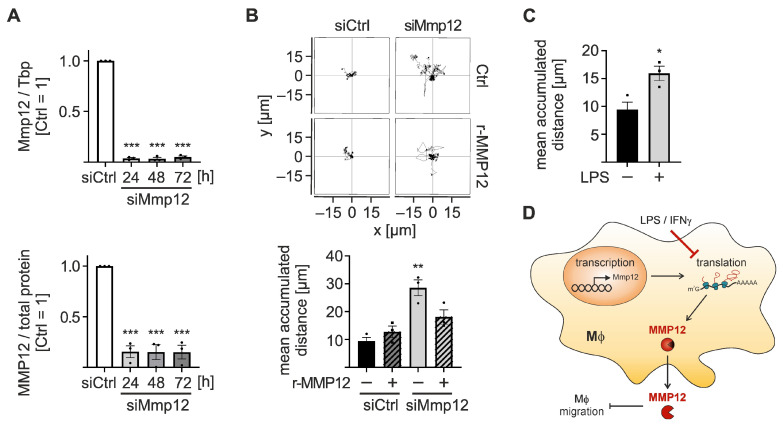
Impact of MMP12 on Mϕ migration. BMDMs were transfected with Mmp12 or Ctrl siRNA (50 nM) for 24, 48, or 72 h. (**A**) Mmp12 mRNA expression was quantified by RT-qPCR analysis, normalized to Tbp, and presented relative to siCtrl-transfected Mϕ (72 h) (*n* = 3; *upper panel*). MMP12 protein expression was analyzed by Western blot analysis, normalized to total protein stain, and presented relative to siCtrl-transfected Mϕ (72 h) (*n* = 3; *lower panel*). (**B**,**C**) Mϕ were seeded 48 h after transfection on Matrigel (0.5×)/elastin (50 µg/mL) coated plates. (**B**) Mϕ were treated with recombinant MMP12 (r-MMP12, 50 ng/mL) 24 h after seeding, and migration was determined by live cell tracking for 24 h and quantified using the ImageJ manual tracking plugin. Representative tracks of siCtrl and siMmp12 in the presence or absence of r-MMP12 are depicted (*upper panel*). The migrated distance of 20 randomly selected cells per field of view was analyzed per replicate (*n* = 3; *lower panel*). (**C**) siCtrl-transfected Mϕ were stimulated with 100 ng/mL LPS and 100 U/mL IFNγ (LPS) 24 h after seeding, and migration was determined by live cell tracking for 24 h (*n* = 3). Data are presented as means ± SEM and were statistically analyzed using two-way ANOVA with Tukey’s multiple comparisons test (**A**,**B**) or unpaired *t*-test (**C**); * *p* < 0.05, ** *p* < 0.01, *** *p* < 0.001 compared to untreated, siCtrl-transfected Mϕ. (**D**) Schematic model of the translational repression of MMP12 in Mϕ by pro-inflammatory LPS/IFNγ stimulation, resulting in reduced MMP12 protein levels and enhanced Mϕ migration in the local environment.

As MMP12 has been suggested to affect macrophage recruitment in an in vivo model of lung inflammation [27], we hypothesized that its expression might play a role in BMDM migration. To assess the impact of MMP12 on migration, we transfected Mϕ with siRNA targeting Mmp12 (siMmp12). This efficiently and stably reduced Mmp12 expression between 24 and 72 h after transfection at the mRNA and protein levels (Figure 6A). Unexpectedly, Mmp12 knockdown increased Mϕ migration on Matrigel/elastin-coated plates by approximately 3-fold compared to siControl-transfected Mϕ (Figure 6B), without altering Mϕ migration on uncoated plates (Figure S4A).

To ensure that the increase in migration was due to reduced MMP12 levels, we supplemented recombinant MMP12 protein (r-MMP12), which efficiently reduced migration in Mmp12-knockdown Mϕ without affecting control Mϕ (Figure 6B). Again, this effect was only observed on Matrigel/elastin-coated plates, not on uncoated plates (Figure S4B). To assess if LPS/IFNγ treatment, which efficiently reduced Mmp12 translation and MMP12 protein, also affects Mϕ migration, we evaluated Mϕ migration in the presence of LPS/IFNγ for 24 h. In line with the reduced MMP12 levels in inflammatory Mϕ, migration specifically increased only on Matrigel/elastin-coated plates (Figure 6C), while migration on uncoated plates remained unaltered (Figure S4C).

Taken together, these findings suggest that the Mϕ elastase MMP12 degrades elastin and thereby prevents the migration of Mϕ along elastin fibers. In an inflammatory context, translational downregulation of MMP12 enhances the migratory capacity of Mϕ, supporting the mobility of Mϕ at the site of inflammation during the early phase of the inflammatory process.

## 3. Discussion

In the present study, we analyzed transcriptional and translational changes in Mϕ upon stimulation with LPS/IFNγ. Further, we assessed changes in the inflammatory RNA and protein expression profiles in Mϕ brought about by the efferocytosis of apoptotic cells. While efferocytosis induced resolution-related transforming growth factor β (TGFβ) signaling and translation-regulatory gene expression signatures in inflammatory Mϕ, we found that the observed transcriptional changes were determined largely by inflammatory stimuli. In contrast, efferocytosis substantially altered de novo synthesized proteins in naïve and inflammatory Mϕ. We further observed a marked, exclusively translational repression of the resolution-associated Mϕ elastase Mmp12 during early inflammation, resulting in a delayed reduction in functional extracellular MMP12 protein levels. Functionally, reduced Mmp12 expression in Mϕ, e.g., during inflammation, enhanced their migratory capacity (Figure 6D).

In contrast to earlier concepts suggesting that mRNA and protein levels are generally closely correlated [28], there is accumulating evidence that transcriptome and translatome changes in response to stimulation diverge substantially in mammalian cells [20,29,30]. For instance, upon infection of primary murine Mϕ with *Leishmania donovani*, a third of the protein-coding mRNAs was differentially translated [31]. Moreover, in tumor-associated Mϕ, gene expression changes during tumor outgrowth appeared to be regulated predominantly via translational regulation [20]. Similarly, we observed marked differences in de novo proteomic changes between the inflammatory response of efferocytic and non-efferocytic macrophages. At the transcriptomic level, regulatory patterns appeared rather similar, suggesting a major contribution of translational changes to the efferocytosis-dependent modulation of inflammatory responses in Mϕ. While efferocytosis-induced translational changes have not been investigated, the close relationship between metabolic and translational changes is well established. For example, glucose or amino acid depletion elicits a stress reaction, which enhances phosphorylation and, thus, inactivates eIF2α, consequently reducing global translation [32,33], while favoring the synthesis of a specific subset of stress response factors such as activating transcription factor 4 (ATF4) [34]. Similarly, targets upregulated by efferocytosis at the RNA level were enriched in autophagy- and metabolism-associated processes, and those upregulated at the de novo protein level were enriched in metabolism-associated terms (Tables S2 and S5). These findings corroborate previous reports suggesting that Mϕ are metabolically overloaded and double their lipid, carbohydrate, protein, and nucleotide content [10,35]. Surprisingly, while translation also appeared to be enriched upon efferocytosis at the de novo proteomic level, this did not correspond to an increase in global translation, as measured via polysomal fractionation analyses. Yet, other studies investigating translational regulation during inflammation also observed a pronounced enrichment in translation-associated candidates, within the translationally regulated targets [36]. Accordingly, we observed translational regulation of numerous ribosomal proteins, such as RPL14, 22, 35, and 8, all known to be translated in a 5′ terminal oligopyrimidine (5′ TOP) element-dependent manner [37]. Further support for the impact of metabolic changes during efferocytosis on translation comes from the observation that mammalian target of rapamycin (mTOR) signaling was enriched in mRNAs upregulated in response to efferocytosis (Tables S2 and S3). mTOR is well established as a crucial signaling node linking metabolism to translation [38,39].

As a side note, the impact of translational regulation on the functional net protein output is likely underestimated in many studies, as transcription and translation are commonly regulated in the same direction. Yet, even in the case of co-directional regulation, translation provides a last control measure to tune expression.

Mmp12 is induced by granulocyte macrophage-colony stimulating factor (GM-CSF) and further enhanced by co-stimulation with TGFβ, IL-1β, and monocyte chemoattractive protein 1 (MCP-1). Additionally, activation of CD40 signaling or anti-inflammatory stimulation with IL-4 induces, while IFNγ attenuates Mmp12 expression [40,41,42]. Here, we observed that while Mmp12 mRNA expression was not affected by LPS/IFNγ, Mmp12 translation was markedly reduced in Mϕ during the early stages of inflammation, reducing levels of secreted functional MMP12 at later time points. While translational regulation of Mmp12 was not previously reported, human Mmp13 mRNA, which is expressed in chronic inflammation, was translationally silenced by an alternatively spliced form of the RNA-binding protein TIAR (T-cell-restricted intracellular antigen-related protein) [43]. Hence, it will be interesting to determine whether a similar mechanism regulates Mmp12 translation or if other RNA-binding proteins are involved. Nevertheless, in line with the stimulatory function of CD40 [40] and the inhibitory function of IFNγ [41], negative regulation of CD40 signaling as well as positive regulation of IFNγ signaling were enriched in LPS/IFNγ-induced genes (Figure S5). Notably, other MMPs, such as Mmp9 and Mmp13, appeared to be induced exclusively by pro-inflammatory stimuli, such as LPS, IL-1β, or TNF [44].

Functionally, MMP12, known as macrophage elastase, belongs to the family of extracellular matrix (ECM) degrading enzymes [45]. It was further proposed as an inflammation resolution factor [46] and shown to alter inflammatory responses by cleaving IFNγ [26], but also via restricting recruitment of leukocytes by cleaving numerous CXC chemokines [47]. However, MMPs also generally act on ECM components, altering cell-matrix interactions and migration [48]. While MMP12 was previously shown to be critical for Mϕ transmigration across intestinal epithelial cell layers in severe colitis [49], we found that MMP12 attenuates Mϕ mobility on Matrigel/elastin-coated surfaces resembling structural features of the ECM [50]. Specifically, reduced Mmp12 expression, achieved via knockdown or inflammatory stimulation, enhanced the Mϕ migratory capacity, which could be overcome by adding exogenous MMP12. The fact that this altered migration phenotype was only observed in the presence of elastin (i.e., the substrate of MMP12) in the scaffold suggests that Mϕ might use elastin fibers as tracks. In fact, collagen fibers were recently proposed as ECM tracks supporting cell migration [51], and nanofiber-based, specific reconstructions of ECM fibers revealed that cell migration properties depend on the exact composition of the fibers [52].

## 4. Conclusions

We provide the first evidence that Mmp12 can be regulated at the translation level. Specifically, we show that Mmp12 is translationally repressed during early inflammation in primary murine Mϕ and increases during the resolution phase. Since MMP12 appears to attenuate migration along elastin fibers, reduced MMP12 levels in an inflammatory environment might enhance the ability of Mϕ to patrol the site of inflammation while simultaneously limiting their trans-epithelial egress. Thus, increasing MMP12 levels at later stages of the inflammatory process might allow for the emigration of macrophages, contributing to the normalization of the local immune cell environment.

## 5. Materials and Methods

### 5.1. Chemicals

All chemicals were obtained from Thermo Fisher Scientific (Dreieich, Germany) unless otherwise indicated. B/B Homodimerizer (dimerizer) was purchased from Takara Bio Europe (Saint-Germain-en-Laye, France), and recombinant murine MMP12 from R&D Systems (Minneapolis, MN, USA).

### 5.2. Cell Culture

Bone marrow-derived Mϕ (BMDMs) were isolated from the femurs of adult male and female C57BL/6 mice (>8 weeks) and differentiated with 20 ng/mL macrophage colony-stimulating factor (M-CSF) and 20 ng/mL granulocyte macrophage colony-stimulating factor (GM-CSF) (both from Immunotools, Friesoythe, Germany) in Dulbecco’s modified eagle medium (DMEM; Thermo Fisher Scientific) supplemented with 10% fetal calf serum (FCS; Capricorn Scientific, Ebsdorfergrund, Germany), 100 U/mL penicillin, and 100 µg/mL streptomycin (both from Thermo Fisher Scientific) over 6 days. NIH-3T3 caspase activatable (CA) cells (clone D10) (kindly provided by Prof. Simone Fulda, Frankfurt, Germany) were cultured in DMEM supplemented with 10% FCS, 100 U/mL penicillin, and 100 µg/mL streptomycin. Cells were incubated at 37 °C in a humidified atmosphere with 5% CO_2_.

### 5.3. Efferocytosis Model

NIH-3T3 CA cells were stimulated for 6 h with the dimerizer (10 nM) to induce apoptosis before adding them to differentiated BMDMs at a 1:5 ratio for 16 h in the continued presence of the dimerizer (10 nM). For inflammatory stimulation, apoptotic cells were removed, BMDMs were washed twice with PBS and then stimulated for 6 h with 100 ng/mL LPS and 100 U/mL murine IFNγ (both from Sigma-Aldrich, Taufkirchen, Germany). CD45^+^ cells were isolated with CD45 MicroBeads by MACS-sorting (Miltenyi Biotec, Bergisch Gladbach, Germany) according to the manufacturer’s instructions for further analyses.

### 5.4. Viability Assay

The viability of NIH-3T3 CA cells and BMDMs was determined by Annexin V (Immunotools) and propidium iodide (PI; Thermo Fisher Scientific) staining and subsequent fluorescence-activated cell sorting (FACS) analysis on a FACSymphony A5 instrument (BD Biosciences, Heidelberg, Germany).

### 5.5. Efferocytosis Assays

To determine efferocytic capacity, apoptotic NIH-3T3 CA cells were stained with pHrodo green (Thermo Fisher Scientific) 6 h after dimerizer (10 nM) treatment, and Mϕ were stained with MitoTracker red. One hour after staining, pHrodo-labeled apoptotic cells were added to MitoTracker-labeled Mϕ at 2:1 and 5:1 ratios. Efferocytic Mϕ were identified as the double-positive cells on an Incucyte^®^ S3 live cell analysis system (Sartorius, Göttingen, Germany).

Alternatively, apoptotic cells were stained with the CFSE Cell Division Tracker kit (Biolegend, San Diego, CA, USA) prior to co-culture with MitoTracker-labeled Mϕ at a 5:1 ratio; efferocytic Mϕ were identified as the double positive cells on a FACSymphony A5 instrument.

### 5.6. RNA Isolation, Reverse Transcription, and Quantitative Polymerase Chain Reaction (RT-qPCR)

RNA was isolated using TRIzol according to the manufacturer’s instructions. RNA was reverse transcribed with the Maxima First Strand cDNA Synthesis Kit, and qPCR analyses were performed using PowerUp SYBR Green Master Mix on QuantStudio 3 and 5 PCR Real-Time Systems (Thermo Fisher Scientific). Primers were obtained from Biomers (Ulm, Germany) and are listed in Table S7.

### 5.7. Western Blot Analysis and ELISA

All reagents used for western blotting were purchased from Sigma-Aldrich unless otherwise indicated. Cells were lysed in lysis buffer (50 mM Tris/HCl, 150 mM NaCl, 5 mM EDTA, 0.5% NP-40; freshly supplemented with 1 mM DTT from Carl Roth, Karlsruhe, Germany; protease inhibitor and phosphatase inhibitor mixes from cOmplete and phosSTOP; Roche, Grenzach-Wyhlen, Germany). Next, 50 µg of total protein was separated by sodium dodecyl-sulfate polyacrylamide gel electrophoresis (SDS-PAGE) and transferred to nitrocellulose membranes (GE Healthcare, Chalfont St Giles, UK). Proteins were detected using a specific antibody for murine MMP12 (R&D Systems, Minneapolis, MN, USA) and appropriate IRDye secondary antibodies (LI-COR Biosciences, Bad Homburg, Germany) and quantified on an Odyssey infrared imaging system (LI-COR Biosciences).

Extracellular quantities of MMP12 were measured in cell culture supernatants using the mouse MMP12 ELISA kit (Abcam, Cambridge, UK) according to the manufacturer’s instructions on an absorption microplate reader (Berthold Technologies, Bad Wildbad, Germany).

### 5.8. Polysomal Fractionation

BMDMs were subjected to polysomal fractionation as described previously [53]. Briefly, cells were incubated with 100 µg/mL cycloheximide (CHX, Carl Roth) for 10 min, washed with PBS/CHX (100 µg/mL), and lysed in 750 µL polysome lysis buffer (140 mM KCl, 20 mM Tris-HCl pH 8.0, 5 mM MgCl_2_, 0.5% NP-40, 0.5 mg/mL heparin, 1 mM DTT, 100 U/mL RNasin from Promega, Mannheim, Germany; 100 µg/mL CHX). After pelleting, 600 µL of the cell lysates were layered onto 11 mL of 10–50% continuous sucrose gradients; 100 µL of the lysate was used for total RNA isolation. Gradients were centrifuged at 35,000 rpm for 2 h at 4 °C without brake using a SW40 rotor (Beckman Coulter, Brea, CA, USA). The gradient was collected into 1-mL fractions using a Gradient Station (BioComp Instruments, Fredericton, Canada), and UV absorbance was measured at 254 nm. RNA was precipitated by adding 1/10 volume of sodium acetate (3 M) and 1 volume of isopropyl alcohol. RNA was further purified using the RNeasy Mini kit (Qiagen, Hilden, Germany) according to the manufacturer’s instructions and pooled into sub-polysomes (fractions 1–4), early polysomes (fractions 5–7), and late polysomes (fractions 8–11). Finally, 500 ng of RNA was reverse-transcribed and quantified by qPCR as described above.

### 5.9. RNA Sequencing

RNA was isolated using the RNA Clean & Concentrator-25 kit (Zymo Research, Freiburg, Germany), and rRNA was depleted from total RNA using the RiboCop rRNA depletion kit (Lexogen, Vienna, Austria) according to the manufacturer’s instructions. RNA quality and quantity were determined using RNA ScreenTape assays on a TapeStation 4150 (Agilent Technologies, Waldbronn, Germany) and Qubit RNA HS Assay Kits on a Qubit 3.0 Fluorometer (Thermo Fisher Scientific). Sequencing libraries were prepared according to the total RNA workflow of the TruSeq Ribo Profile (Mammalian) Library Prep Kit (Illumina, San Diego, CA, USA). Briefly, rRNA-depleted RNA was heat-fragmented (94 °C, 25 min) and end-repaired. Thereafter, the 3′ adapter was ligated, and reverse transcription was performed. After PAGE purification, cDNA was circularized, and PCR amplified. The quality of cDNA libraries was assessed using HS-D1000 ScreenTape assays on a TapeStation 4150, and quantities were measured using Qubit dsDNA HS Assay Kits. Libraries were sequenced (single end, 50 cycles) using a P2 100-cycle kit on a NextSeq 2000 instrument (Illumina).

Data processing was performed using cutadapt for adapter trimming [54], bowtie2 for rRNA removal [55], and STAR for mapping the samples to the mouse genome (mm39) [56]. Transcript counts were determined using htseq-count with default parameters [57]. Differentially expressed genes were determined using DESeq2 in R [58]. *P* values were adjusted by Benjamini-Hochberg FDR correction. Differentially regulated transcripts were visualized with ComplexHeatmaps R package [59] by subjecting read counts to row-wise *z*-score normalization and grouping by *k*-means clustering. To identify enriched functional annotation clusters, transcripts were analyzed using the Database for Annotation, Visualization and Integrated Discovery (DAVID) against the gene set “GOTERM_BP_ALL” [23,24]. A list of all detected transcripts (basemean > 0, for all conditions) served as the background data set.

### 5.10. De Novo Proteomics (Multiplexed Enhanced Protein Dynamics (mePROD) Proteomics)

For pulse labeling experiments, BMDMs were co-cultured with apoptotic NIH-3T3 CA cells, as described before. After washing two times with PBS, cells were incubated with DMEM medium for SILAC containing 100 µg/mL Arg10 and 100 µg/mL Lys8 (both from Cambridge Isotope Laboratories, Tewksbury, MA, USA) and stimulated with 100 ng/mL LPS and 100 U/mL IFNγ for 6 h. To obtain a fully labeled sample, BMDMs were cultured in DMEM for SILAC for two weeks. For cell harvest, CD45^+^ cells were isolated out of co-cultures as described before. A fully labeled sample and an unlabeled sample were washed three times with PBS. After pelleting, 1 × 10^6^ cells were flash-frozen in liquid nitrogen. Sample preparation for LC-MS, mass spectrometry and subsequent data analysis and statistics were performed as described previously [25,60,61].

### 5.11. Migration Assay

Differentiated BMDMs were transfected using HiPerFect transfection reagent (Qiagen) with 50 nM Mmp12 (ON-TARGET plus Mouse Mmp12 siRNA—SMARTpool) or Ctrl siRNA (ON-TARGETplus Non-targeting Pool; both Dharmacon, Lafayette, LA, USA) for 48 h and seeded in 24-well culture plates coated with 0.5 × Matrigel supplemented with 50 µg/mL mouse elastin (Sigma-Aldrich). After 24 h, the cells were imaged every 10 min for 24 h at 37 °C and 5% CO_2_ using a Cell Observer microscope (Carl Zeiss, Oberkochen, Germany). Analysis of Mϕ migration was performed using the ImageJ (v. 2.0.0-rc-56) manual tracking plugin. Twenty randomly chosen cells per field of view were tracked per condition for each replicate. Migration plots were generated for 20 tracks using the Chemotaxis and Migration Tool from ibidi (Gräfelfing, Germany).

### 5.12. Statistical Analysis

Statistics were performed with GraphPad Prism v9.3.1 (GraphPad Software, San Diego, CA, USA). Data are reported as means ± SEM of at least three independent experiments. Normal distribution was assessed using the Shapiro-Wilk test. Statistically significant differences were calculated using two-way ANOVA with Tukey’s multiple comparisons test or Students *t*-test.

## Data Availability

The sequencing data presented in this study are available in GEO accession number GSE245834.

## Supplementary Materials

The following supporting information can be downloaded at: https://www.mdpi.com/article/10.3390/ijms242316981/s1.

## References

[1] Fioranelli M., Roccia M.G., Flavin D., Cota L. (2021). Regulation of Inflammatory Reaction in Health and Disease. Int. J. Mol. Sci..

[2] Dalli J., Serhan C.N. (2017). Pro-Resolving Mediators in Regulating and Conferring Macrophage Function. Front. Immunol..

[3] Fullerton J.N., Gilroy D.W. (2016). Resolution of inflammation: A new therapeutic frontier. Nat. Rev. Drug Discov..

[4] Savill J.S., Wyllie A.H., Henson J.E., Walport M.J., Henson P.M., Haslett C. (1989). Macrophage phagocytosis of aging neutrophils in inflammation. Programmed cell death in the neutrophil leads to its recognition by macrophages. J. Clin. Investig..

[5] Gautier E.L., Ivanov S., Lesnik P., Randolph G.J. (2013). Local apoptosis mediates clearance of macrophages from resolving inflammation in mice. Blood.

[6] Doran A.C., Yurdagul A., Tabas I. (2020). Efferocytosis in health and disease. Nat. Rev. Immunol..

[7] Razi S., Yaghmoorian Khojini J., Kargarijam F., Panahi S., Tahershamsi Z., Tajbakhsh A., Gheibihayat S.M. (2023). Macrophage efferocytosis in health and disease. Cell Biochem. Funct..

[8] Elliott M.R., Koster K.M., Murphy P.S. (2017). Efferocytosis Signaling in the Regulation of Macrophage Inflammatory Responses. J. Immunol..

[9] Fadok V.A., Bratton D.L., Konowal A., Freed P.W., Westcott J.Y., Henson P.M. (1998). Macrophages that have ingested apoptotic cells in vitro inhibit proinflammatory cytokine production through autocrine/paracrine mechanisms involving TGF-beta, PGE2, and PAF. J. Clin. Investig..

[10] Schilperoort M., Ngai D., Sukka S.R., Avrampou K., Shi H., Tabas I. (2023). The role of efferocytosis-fueled macrophage metabolism in the resolution of inflammation. Immunol. Rev..

[11] Collins G., Souza Carvalho J., de Gilroy D.W. (2023). The translation potential of harnessing the resolution of inflammation. J. Allergy Clin. Immunol..

[12] Saas P., Vetter M., Maraux M., Bonnefoy F., Perruche S. (2022). Resolution therapy: Harnessing efferocytic macrophages to trigger the resolution of inflammation. Front. Immunol..

[13] Zhang J., Ding W., Zhao M., Liu J., Xu Y., Wan J., Wang M. (2022). Mechanisms of efferocytosis in determining inflammation resolution: Therapeutic potential and the association with cardiovascular disease. Br. J. Pharmacol..

[14] Das A.S., Basu A., Kumar R., Borah P.K., Bakshi S., Sharma M., Duary R.K., Ray P.S., Mukhopadhyay R. (2020). Post-transcriptional regulation of C-C motif chemokine ligand 2 expression by ribosomal protein L22 during LPS-mediated inflammation. FEBS J..

[15] Naqvi R.A., Gupta M., George A., Naqvi A.R. (2022). MicroRNAs in shaping the resolution phase of inflammation. Semin. Cell Dev. Biol..

[16] Rappl P., Brüne B., Schmid T. (2021). Role of Tristetraprolin in the Resolution of Inflammation. Biology.

[17] Piccirillo C.A., Bjur E., Topisirovic I., Sonenberg N., Larsson O. (2014). Translational control of immune responses: From transcripts to translatomes. Nat. Immunol..

[18] Bosurgi L., Cao Y.G., Cabeza-Cabrerizo M., Tucci A., Hughes L.D., Kong Y., Weinstein J.S., Licona-Limon P., Schmid E.T., Pelorosso F. (2017). Macrophage function in tissue repair and remodeling requires IL-4 or IL-13 with apoptotic cells. Science.

[19] Rappl P., Rösser S., Maul P., Bauer R., Huard A., Schreiber Y., Thomas D., Geisslinger G., Jakobsson P.-J., Weigert A. (2021). Inhibition of mPGES-1 attenuates efficient resolution of acute inflammation by enhancing CX3CL1 expression. Cell Death Dis..

[20] Bartish M., Tong D., Pan Y., Wallerius M., Liu H., Ristau J., Souza Ferreira S., de Wallmann T., van Hoef V., Masvidal L. (2020). MNK2 governs the macrophage antiinflammatory phenotype. Proc. Natl. Acad. Sci. USA.

[21] Knuth A.-K., Huard A., Naeem Z., Rappl P., Bauer R., Mota A.C., Schmid T., Fleming I., Brüne B., Fulda S. (2021). Apoptotic Cells induce Proliferation of Peritoneal Macrophages. Int. J. Mol. Sci..

[22] Young S.K., Wek R.C. (2016). Upstream Open Reading Frames Differentially Regulate Gene-specific Translation in the Integrated Stress Response. J. Biol. Chem..

[23] Da Huang W., Sherman B.T., Lempicki R.A. (2009). Systematic and integrative analysis of large gene lists using DAVID bioinformatics resources. Nat. Protoc..

[24] Sherman B.T., Hao M., Qiu J., Jiao X., Baseler M.W., Lane H.C., Imamichi T., Chang W. (2022). DAVID: A web server for functional enrichment analysis and functional annotation of gene lists (2021 update). Nucleic Acids Res..

[25] Klann K., Tascher G., Münch C. (2020). Functional Translatome Proteomics Reveal Converging and Dose-Dependent Regulation by mTORC1 and eIF2α. Mol. Cell.

[26] Dufour A., Bellac C.L., Eckhard U., Solis N., Klein T., Kappelhoff R., Fortelny N., Jobin P., Rozmus J., Mark J. (2018). C-terminal truncation of IFN-γ inhibits proinflammatory macrophage responses and is deficient in autoimmune disease. Nat. Commun..

[27] Hautamaki R.D., Kobayashi D.K., Senior R.M., Shapiro S.D. (1997). Requirement for macrophage elastase for cigarette smoke-induced emphysema in mice. Science.

[28] Crick F. (1970). Central dogma of molecular biology. Nature.

[29] Schott J., Reitter S., Philipp J., Haneke K., Schäfer H., Stoecklin G. (2014). Translational regulation of specific mRNAs controls feedback inhibition and survival during macrophage activation. PLoS Genet..

[30] Tebaldi T., Re A., Viero G., Pegoretti I., Passerini A., Blanzieri E., Quattrone A. (2012). Widespread uncoupling between transcriptome and translatome variations after a stimulus in mammalian cells. BMC Genom..

[31] Chaparro V., Leroux L.-P., Masvidal L., Lorent J., Graber T.E., Zimmermann A., Arango Duque G., Descoteaux A., Alain T., Larsson O. (2020). Translational profiling of macrophages infected with Leishmania donovani identifies mTOR- and eIF4A-sensitive immune-related transcripts. PLoS Pathog..

[32] Ashe M.P., de Long S.K., Sachs A.B. (2000). Glucose depletion rapidly inhibits translation initiation in yeast. Mol. Biol. Cell.

[33] Zhang P., McGrath B.C., Reinert J., Olsen D.S., Lei L., Gill S., Wek S.A., Vattem K.M., Wek R.C., Kimball S.R. (2002). The GCN2 eIF2alpha kinase is required for adaptation to amino acid deprivation in mice. Mol. Cell. Biol..

[34] Vattem K.M., Wek R.C. (2004). Reinitiation involving upstream ORFs regulates ATF4 mRNA translation in mammalian cells. Proc. Natl. Acad. Sci. USA.

[35] Han C.Z., Ravichandran K.S. (2011). Metabolic connections during apoptotic cell engulfment. Cell.

[36] Ceppi M., Clavarino G., Gatti E., Schmidt E.K., de Gassart A., Blankenship D., Ogola G., Banchereau J., Chaussabel D., Pierre P. (2009). Ribosomal protein mRNAs are translationally-regulated during human dendritic cells activation by LPS. Immunome Res..

[37] Cockman E., Anderson P., Ivanov P. (2020). TOP mRNPs: Molecular Mechanisms and Principles of Regulation. Biomolecules.

[38] Liu B., Qian S.-B. (2011). Translational regulation in nutrigenomics. Adv. Nutr..

[39] Ma X.M., Blenis J. (2009). Molecular mechanisms of mTOR-mediated translational control. Nat. Rev. Mol. Cell Biol..

[40] Wu L., Fan J., Matsumoto S.I., Watanabe T. (2000). Induction and regulation of matrix metalloproteinase-12 by cytokines and CD40 signaling in monocyte/macrophages. Biochem. Biophys. Res. Commun..

[41] Jost M.M., Ninci E., Meder B., Kempf C., van Royen N., Hua J., Berger B., Hoefer I., Modolell M., Buschmann I. (2003). Divergent effects of GM-CSF and TGFbeta1 on bone marrow-derived macrophage arginase-1 activity, MCP-1 expression, and matrix metalloproteinase-12: A potential role during arteriogenesis. FASEB J..

[42] Shimizu K., Shichiri M., Libby P., Lee R.T., Mitchell R.N. (2004). Th2-predominant inflammation and blockade of IFN-γ signaling induce aneurysms in allografted aortas. J. Clin. Investig..

[43] Mazumder B., Li X., Barik S. (2010). Translation control: A multifaceted regulator of inflammatory response. J. Immunol..

[44] Castrillo A., Joseph S.B., Marathe C., Mangelsdorf D.J., Tontonoz P. (2003). Liver X receptor-dependent repression of matrix metalloproteinase-9 expression in macrophages. J. Biol. Chem..

[45] Shapiro S.D. (1998). Matrix metalloproteinase degradation of extracellular matrix: Biological consequences. Curr. Opin. Cell Biol..

[46] Mouton A.J., Rivera Gonzalez O.J., Kaminski A.R., Moore E.T., Lindsey M.L. (2018). Matrix metalloproteinase-12 as an endogenous resolution promoting factor following myocardial infarction. Pharmacol. Res..

[47] Dean R.A., Cox J.H., Bellac C.L., Doucet A., Starr A.E., Overall C.M. (2008). Macrophage-specific metalloelastase (MMP-12) truncates and inactivates ELR+ CXC chemokines and generates CCL2, -7, -8, and -13 antagonists: Potential role of the macrophage in terminating polymorphonuclear leukocyte influx. Blood.

[48] Murray M.Y., Birkland T.P., Howe J.D., Rowan A.D., Fidock M., Parks W.C., Gavrilovic J. (2013). Macrophage migration and invasion is regulated by MMP10 expression. PLoS ONE.

[49] Nighot M., Ganapathy A.S., Saha K., Suchanec E., Castillo E.F., Gregory A., Shapiro S., Ma T., Nighot P. (2021). Matrix Metalloproteinase MMP-12 Promotes Macrophage Transmigration Across Intestinal Epithelial Tight Junctions and Increases Severity of Experimental Colitis. J. Crohns Colitis.

[50] Valdoz J.C., Johnson B.C., Jacobs D.J., Franks N.A., Dodson E.L., Sanders C., Cribbs C.G., van Ry P.M. (2021). The ECM: To Scaffold, or Not to Scaffold, That Is the Question. Int. J. Mol. Sci..

[51] Kassianidou E., Probst D., Jäger J., Lee S., Roguet A.-L., Schwarz U.S., Kumar S. (2019). Extracellular Matrix Geometry and Initial Adhesive Position Determine Stress Fiber Network Organization during Cell Spreading. Cell Rep..

[52] Estabridis H.M., Jana A., Nain A., Odde D.J. (2018). Cell Migration in 1D and 2D Nanofiber Microenvironments. Ann. Biomed. Eng..

[53] Rübsamen D., Blees J.S., Schulz K., Döring C., Hansmann M.-L., Heide H., Weigert A., Schmid T., Brüne B. (2012). IRES-dependent translation of egr2 is induced under inflammatory conditions. RNA.

[54] Martin M. (2011). Cutadapt removes adapter sequences from high-throughput sequencing reads. EMBnet J..

[55] Langmead B., Salzberg S.L. (2012). Fast gapped-read alignment with Bowtie 2. Nat. Methods.

[56] Dobin A., Davis C.A., Schlesinger F., Drenkow J., Zaleski C., Jha S., Batut P., Chaisson M., Gingeras T.R. (2013). STAR: Ultrafast universal RNA-seq aligner. Bioinformatics.

[57] Anders S., Pyl P.T., Huber W. (2015). HTSeq—A Python framework to work with high-throughput sequencing data. Bioinformatics.

[58] Love M.I., Huber W., Anders S. (2014). Moderated estimation of fold change and dispersion for RNA-seq data with DESeq2. Genome Biol..

[59] Gu Z., Eils R., Schlesner M. (2016). Complex heatmaps reveal patterns and correlations in multidimensional genomic data. Bioinformatics.

[60] Klann K., Münch C. (2020). Instrument Logic Increases Identifications during Multiplexed Translatome Measurements. Anal. Chem..

[61] Schäfer J.A., Bozkurt S., Michaelis J.B., Klann K., Münch C. (2022). Global mitochondrial protein import proteomics reveal distinct regulation by translation and translocation machinery. Mol. Cell.

