# Supplementary material for: Mmp12 Is Translationally Regulated in Macrophages during the Course of Inflammation
Source: Int J Mol Sci. 2023 Nov 30;24(23):16981. doi: 10.3390/ijms242316981 (PMC10707645; doi:10.3390/ijms242316981)
Supplement: Supplementary file 1 [file ijms-24-16981-s001.zip › Kuntschar et al - Supplementary Information.pdf]

SUPPLEMENTARY INFORMATION

Supplementary Figures

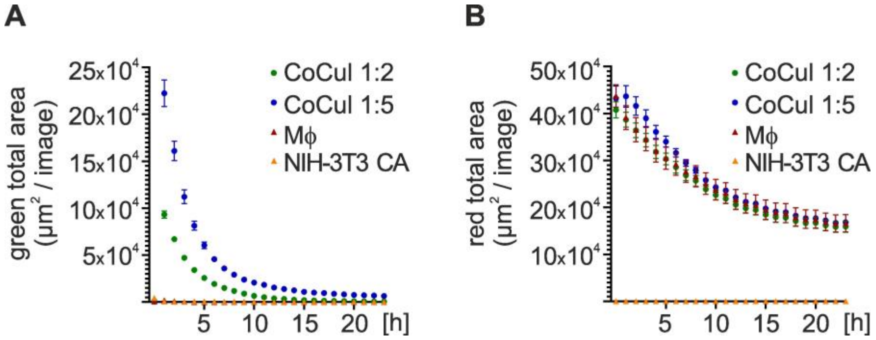

Figure S1. Efferocytosis assay of BMDM co-cultured with apoptotic NIH-3T3 CA cells. BMDM were stained with MitoTracker red and apoptotic NIH-3T3 CA cells (after 6 h treatment with 10 nM dimerizer) were stained with pHrodo for 1 h prior to co-culture at a ratio of 1:2 or 1:5. Green (**A**) and red (**B**) cells were measured with Incucyte live cell analysis system for 24 h (n = 3). Data are presented as means ± SEM.

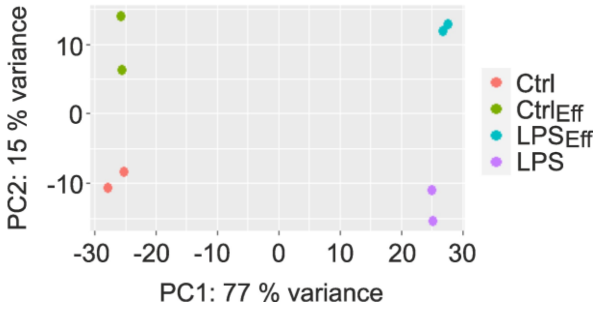

Figure S2. Principal component analysis of total RNA-seq data. BMDM were co-cultured with or without apoptotic NIH-3T3 CA cells (Eff) (after 6 h dimerizer treatment) at a ratio of 1:5 for 16 h prior to stimulation with 100 ng/mL LPS and 100 U/mL IFNγ for 6 h (LPS). $CD45^{+}$ Mϕ were purified by MACS-sorting followed by total RNA-seq analysis (n = 2). Principal component analysis of normalized read counts of all genes is shown.

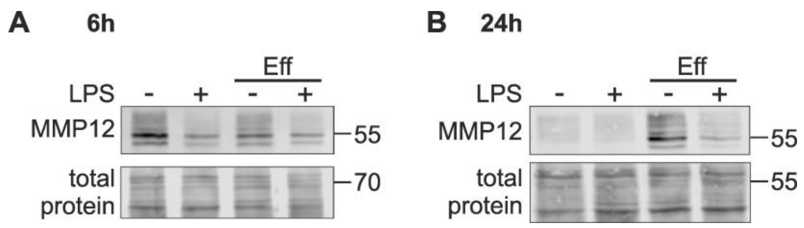

Figure S3. MMP12 protein expression in inflammatory Mϕ. BMDM were co-cultured with or without apoptotic NIH-3T3 CA cells (Eff) (after 6 h dimerizer treatment) at a ratio of 1:5 for 16 h prior to stimulation with 100 ng/mL LPS and 100 U/mL IFNγ for 6 (**A**) or 24 h (**B**) (LPS). For further analyses, $CD45^+$ Mϕ were purified by MACS-sorting. MMP12 protein expression was analyzed by Western blot analysis. Representative blots of at least 5 independent experiments are shown.

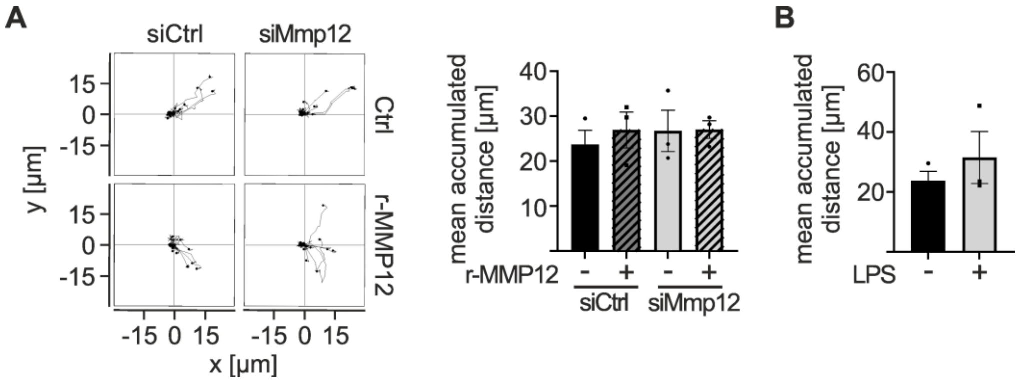

Figure S4. Mϕ migration on uncoated plates. (**A**) BMDM were transfected with Mmp12 or Ctrl siRNA (20 µM) for 48 h and then seeded on uncoated cell culture plates. 24 h after seeding, Mϕ were treated with recombinant murine MMP12 (r-MMP12, 50 ng/mL), migration was determined by live cell tracking for 24 h, and quantified using ImageJ manual tracking plugin. Representative tracks for siCtrl and siMmp12 in the presence or absence of r-MMP12 are depicted (*left panel*). Migrated distance of 20 randomly selected cells per field of view were analyzed per replicate (n = 3; *right panel*). (**B**) 24 h after seeding, siCtrl-transfected Mϕ on uncoated cell culture plates, were stimulated with 100 ng/mL LPS and 100 U/mL IFNγ (LPS) and migration was determined by live cell tracking for 24 h (n = 3). Data are presented as means ± SEM and were statistically analyzed using two-way ANOVA with Tukey's multiple comparisons test (A, B) or unpaired t-test (C).

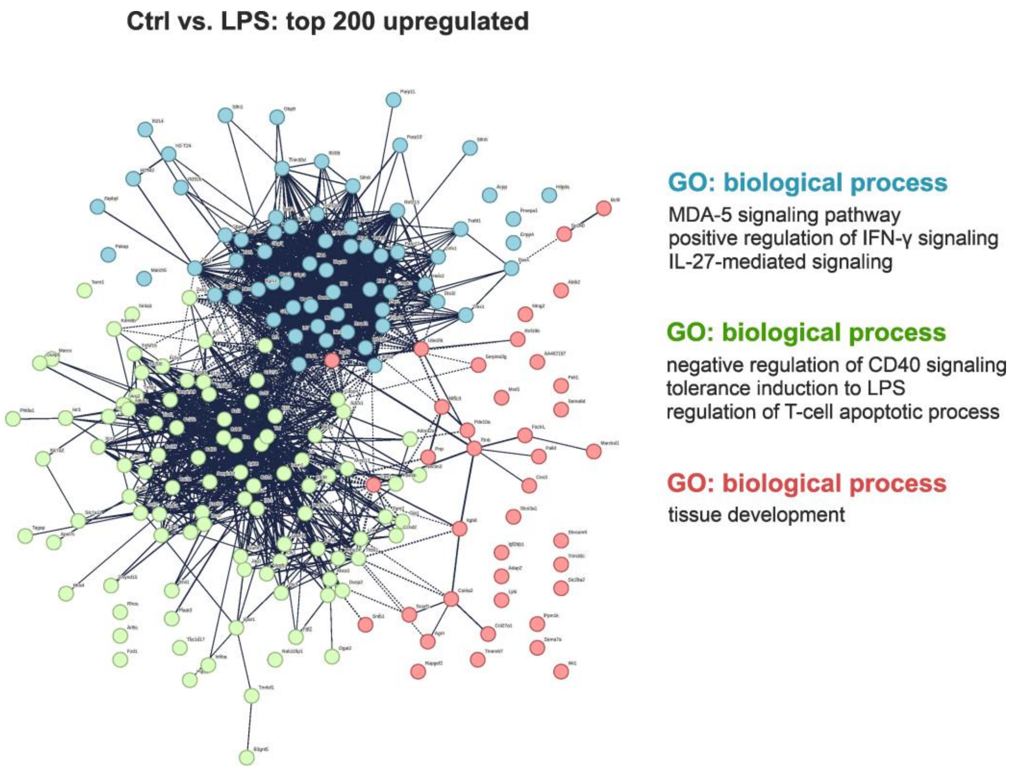

Figure S5. String analysis of the top 200 upregulated mRNAs in Mϕ stimulated with LPS and IFNγ for 6 h.

Supplementary Tables

Table S7. Primers.

**primer**	**forward**	**reverse**
Gapdh	AGG TCG GTG TGA ACG GAT TTG	TGT AGA CCA TGT AGT TGA GGT CA
Il-1β	TGA AAT GCC ACC TTT TGA CA	AGC TTC TCC ACA GCC AC AAT
Il-6	TAG TCC TTC CTA CCC CAA TTT CC	TTG GTC CTT AGC CAC TCC TTC
Il-10	GCT CTT ACT GAC TGG CAT GAG	CGC AGC TCT AGG AGC ATG TG
Mmp12	CTG CTC CCA TGA ATG ACA GTG	AGT TGC TTC TAG CCC AAA GAA
Tbp	CTG ACC ACT GCA CCG TTG CCA	GAC TGC AGC AAA TCG CTT GGG A
